# Dual Chyle Leak: A Case of Chylothorax With Chylous Ascites

**DOI:** 10.7759/cureus.97285

**Published:** 2025-11-19

**Authors:** Fathimathu Afrah, Vengadakrishna Krishnamoorthy, Suja Lakshmanan, Saajid Anwar

**Affiliations:** 1 General Medicine, Sri Ramachandra Institute of Higher Education and Research, Chennai, IND; 2 Internal Medicine, Sri Ramachandra Institute of Higher Education and Research, Chennai, IND

**Keywords:** chyle, chylothorax, thoracic duct, thoracic duct embolization, venous thrombosis

## Abstract

Chylothorax is a rare and often insidious disorder, characterised by the accumulation of chyle in the pleural cavity. It results in substantial diagnostic and therapeutic challenges. We report the case of a 56-year-old male with a two-year history of chronic liver disease on treatment, who presented with complaints of breathlessness and abdominal distension for 10 days. Evaluation demonstrated chylothorax, chylous ascites, and a multi-thrombotic state. The underlying etiology was multifactorial, including a thoracic duct leak at the T4-T5 vertebral level. Thoracic duct embolisation was performed, which resulted in a marked reduction in pleural chyle accumulation and significant symptomatic relief.

## Introduction

Chylothorax is the presence of chyle within the pleural space. Chyle is a lipid-rich, milky fluid produced during digestion and contains triglycerides (TGLs), fat-soluble vitamins, lymphocytes, and immunoglobulins. Although uncommon, it accounts for roughly 3% of all pleural effusions [1].

The thoracic duct originates from the cisterna chyli, the confluence of intra-abdominal lymphatics (hepatic, mesenteric, retroperitoneal, and lower limb). It terminates at the junction of the left subclavian and internal jugular veins. Chyle flow increases after fat ingestion, particularly long-chain TGLs. Any disruption, injury, or obstruction of this pathway may result in chylothorax [2].

Disruption or obstruction of the thoracic duct may result from trauma, such as surgical injury, blunt trauma, or central line placements. It can also be due to non-traumatic causes, like malignancies, lymphatic anomalies, and central venous thrombosis. Particular non-traumatic examples include advanced liver cirrhosis with portal hypertension, which can notably increase splanchnic lymphatic flow and pressure. Anatomical variations can further affect the side and severity of chylothorax, depending on where the injury or obstruction occurs [3].

In the present case, several mechanisms contributed to chylothorax. The complexity of diagnosis, the need for multidisciplinary care, and the outcomes following interventional radiology are discussed.

## Case presentation

A 56-year-old man with a history of chronic liver disease (diagnosed in 2023, on medical management) presented with abdominal distension for 10 days. The patient also had modified Medical Research Council (mMRC) Grade 3 breathlessness for 10 days.

Clinical findings

Stable Vital Signs

Alopecia was present; there were prominent veins over the neck, thorax, flanks, and interscapular region. There was no facial edema or flushing of the face. Respiratory system examination revealed decreased breath sounds, vocal fremitus, and vocal resonance over the right mammary, infra-axillary, infrascapular, and interscapular areas. Abdominal examination revealed uniform distension of the abdomen, with shifting dullness present and no organomegaly. Other systems examination was unremarkable.

Investigation

**Figure 1:**
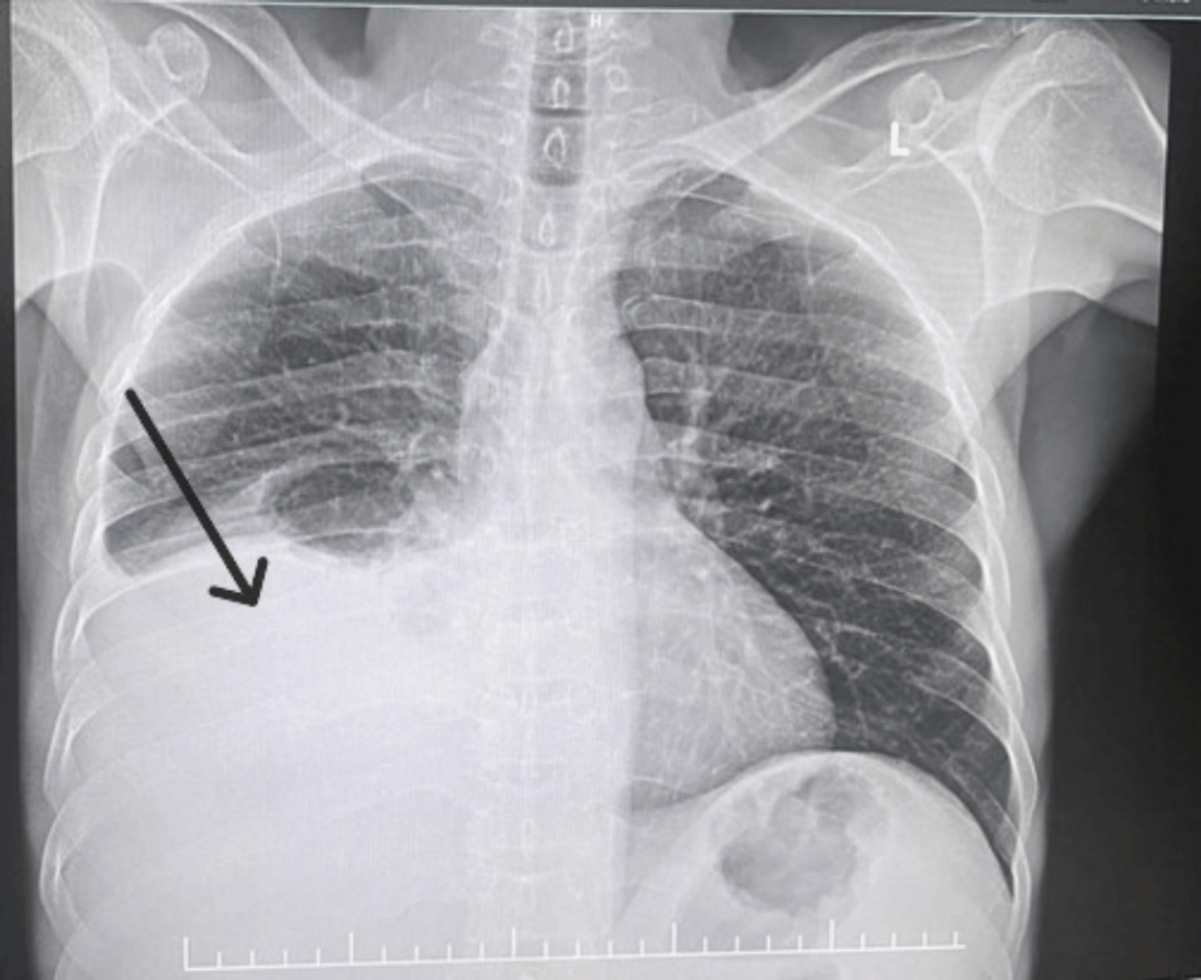
Chest X-ray showing right moderate pleural effusion (arrow) Blunting of the right costophrenic angle and homogeneous opacity of the lower and middle zones.

Routine investigations are listed in Table 1. ECG showed a normal sinus rhythm. Chest X-ray showed blunting of the right costophrenic angle and homogeneous opacity of the lower and middle zones, indicating moderate right pleural effusion, as shown in Figure 1. Nearly 1.2 litres of pleural fluid was tapped, and the pleural fluid analysis showed features of chylous effusion with high TGL levels, as mentioned in Table 2.

**Table 1:** Routine investigation Serial LFT and INR were normal. Hb, Hemoglobin; TLC, Total leukocyte count; BUN, Blood urea nitrogen; LFT, Liver function test; INR, International normalized ratio

Lab Investigations	Values	Reference
Hb	8.7 g/dL	13 - 17 g/dL
TLC	5700 cells/cumm	4000 - 11000 cells/cumm
Platelets	1 lakh/cumm	1.4 - 4.5 lakh/cumm
BUN	10 mg/dL	6 - 20 mg/dL
Creatinine	1.6 mg/dL	0.5 - 0.9 mg/dL

**Table 2:** Pleural fluid analysis Normal pleural fluid contains 98% MN (23% lymphocytes and 75% macrophages/monocytes) and less than 2% PMN. WBC, White blood cell; MN, Mononuclear cells (lymphocytes and monocytes); PMN, Polymorphonuclear cells (neutrophils); ADA, Adenosine deaminase; LDH, Lactate dehydrogenase

Laboratory Investigations	Result	Reference Values
WBC - Pleural fluid	163 cells/cumm	500-1000 cells/cumm
MN cell count	151 cells/cumm	490-980 cells/cumm
PMN cell count	12 cells/cumm	<20 cells/cumm
Triglycerides	1666 mg/dL	<150 mg/dL
ADA	6.4 U/L	<40 U/L
Glucose	121 mg/dL	70-110 mg/dL
Cholesterol	40 mg/dL	<45 mg/dL
Pleural fluid LDH	157 U/L	<200 U/L
Serum LDH	326 U/L	135-225 U/L
Pleural fluid protein	2.42 g/dL	<3 g/dL

Pleural fluid cytology was negative for malignant cells. Pleural fluid acid-fast bacilli (AFB), TB GeneXpert, and culture were negative. For further work-up, a contrast-enhanced computed tomography (CECT) thorax was performed, revealing a severe right pleural effusion with collapse of the right lower lobe and right middle lobe. It also showed acute thrombosis of the right brachiocephalic vein, right subclavian vein, and lower part of the jugular veins, as well as chronic thrombosis of the left jugular and brachiocephalic veins with minimal recanalisation.

**Figure 2:**
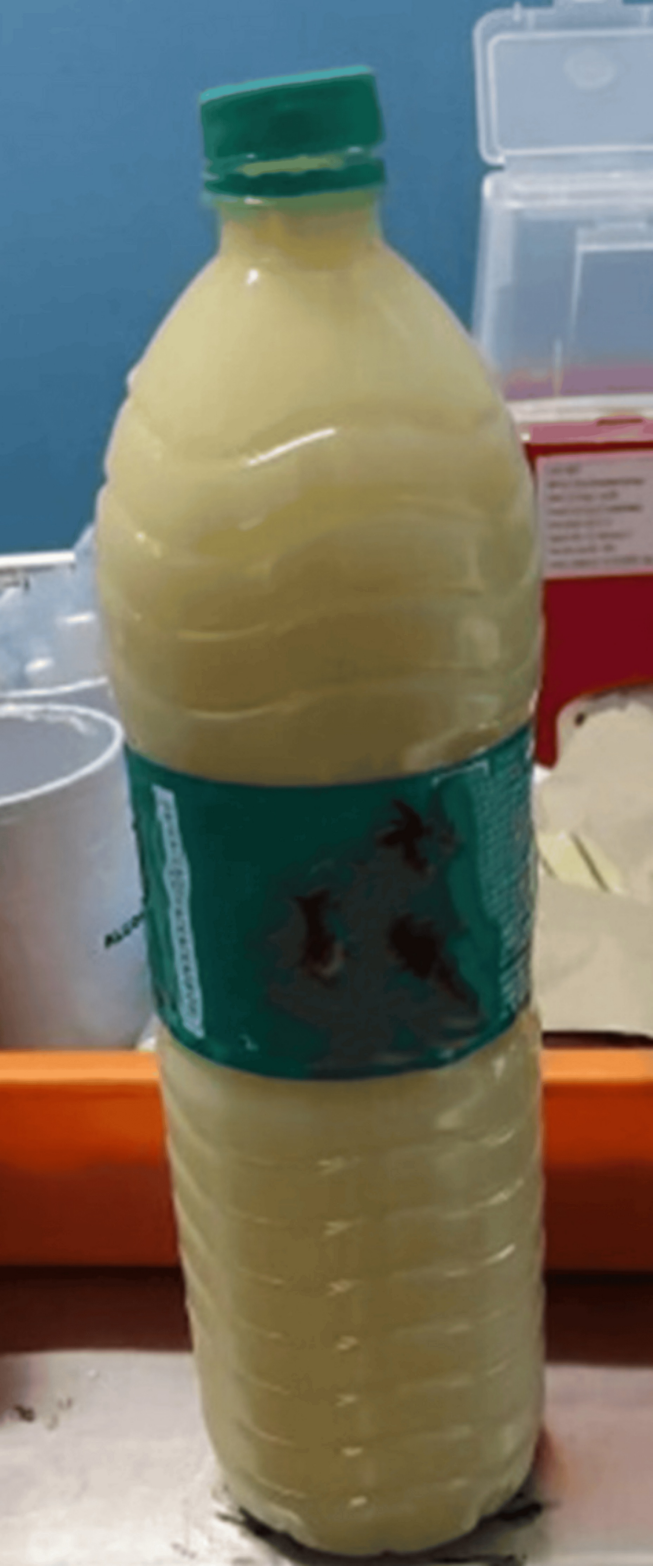
Drained chylous ascitic fluid Milky white ascitic fluid was drained, which on analysis revealed high SAAG, low protein, mononuclear predominance, and a high TGL level, suggesting chylous ascites. SAAG, Serum-ascites albumin gradient; TGL, Triglyceride

As the patient exhibited a tense, distended abdomen, ascitic fluid tapping was done. Ascitic fluid analysis showed a high SAAG (serum-ascites albumin gradient), low ascitic fluid protein, and chylous collection (Figure 2). Ascitic fluid white blood cell (WBC) was 150/µL (monocyte-predominant). Ascitic fluid TGLs were 620 mg/dL (high), and the cytology was negative.

**Figure 3:**
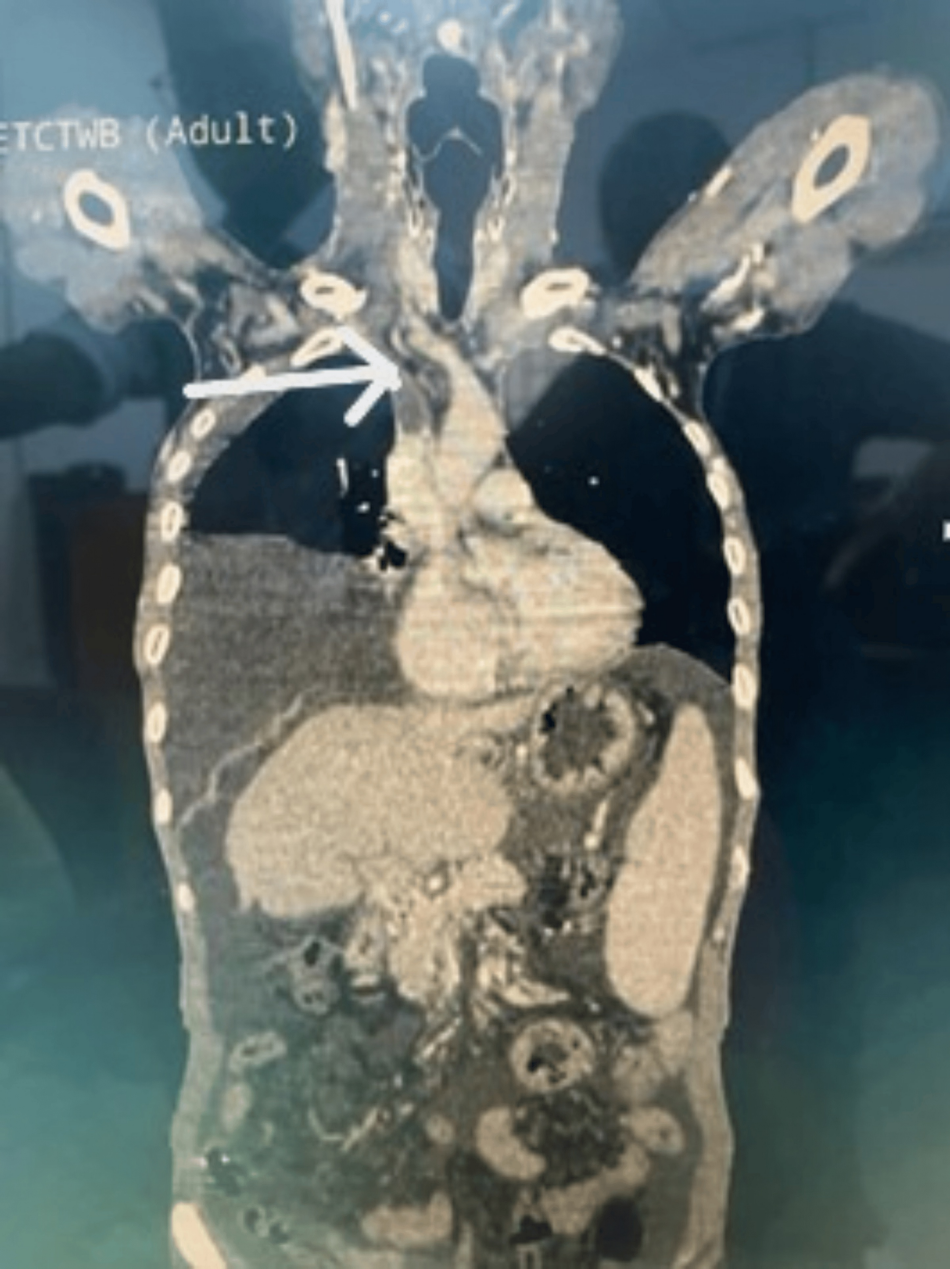
PET-CT showing right pleural effusion with subacute thrombosis of the right brachiocephalic vein, jugular vein, and proximal SVC (arrow) SVC, Superior vena cava; PET-CT, Positron emission tomography-computed tomography

In view of chylous collection and a multiple thrombotic state, malignancy was suspected, and positron emission tomography-computed tomography (PET-CT) was performed. PET-CT reported chronic liver parenchymal disease with chronic portal, mesenteric, and splenic vein thrombosis with collateral formation. It revealed the presence of a massive right pleural effusion; chronic thrombosis of the left internal jugular, brachiocephalic, and subclavian veins; and subacute thrombosis of the right brachiocephalic, jugular, and proximal superior vena cava (SVC) (Figure 3).

Management

**Figure 4:**
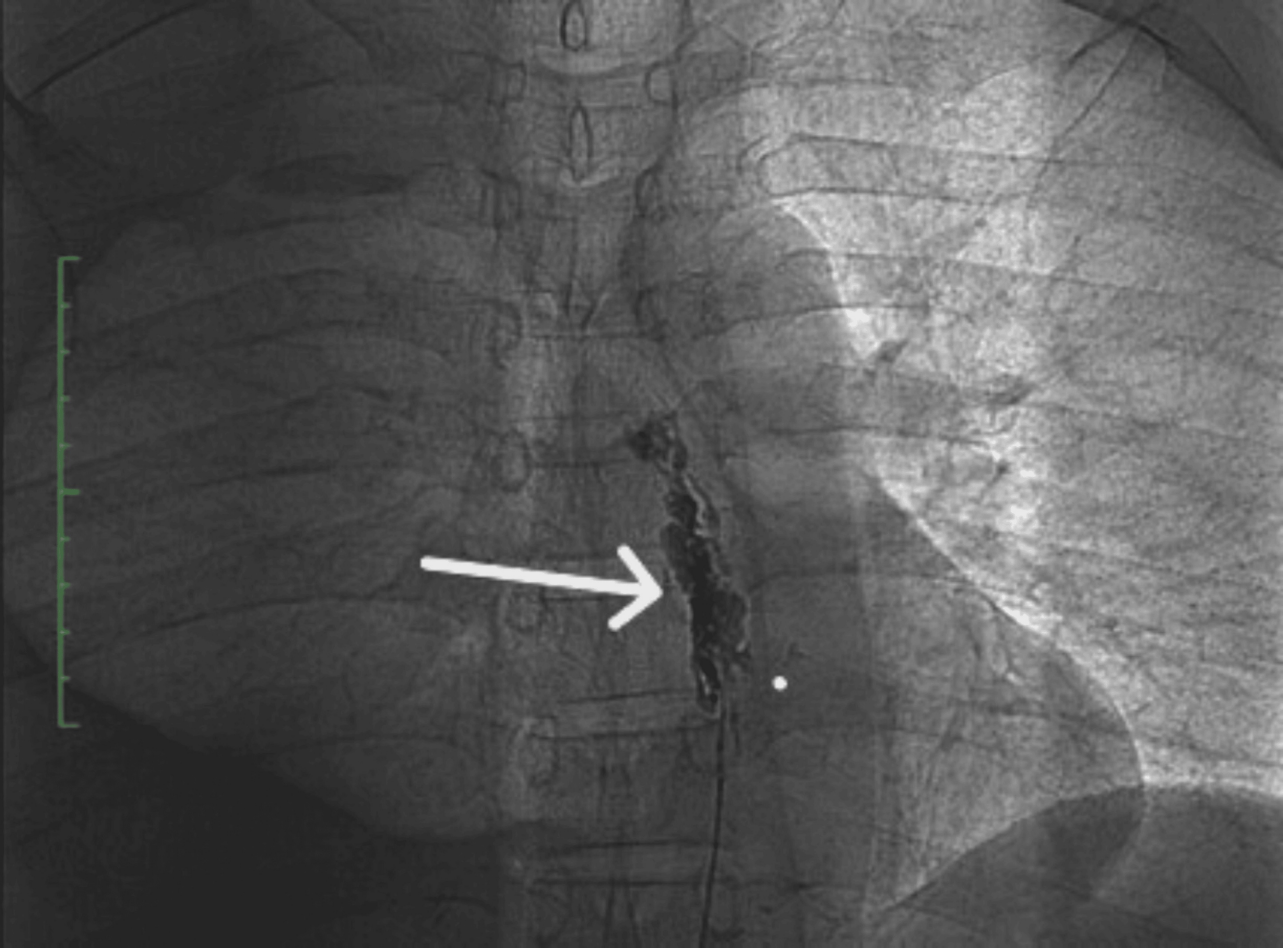
Cisterna chyli during direct intranodal lymphagiography (arrow)

The patient was started on anticoagulation, supplemented with a high-protein, low-fat diet, and discharged. Within one month, he re-presented with recurrent breathlessness. Repeat chest X-ray confirmed massive effusion. In view of repeated collection, magnetic resonance (MR) lymphangiography was performed, revealing no focal leak from the thoracic duct or cisterna chyli. Thoracic duct obstruction at the veno-lymphatic junction (left subclavian vein occlusion), elevated central venous pressure due to SVC occlusion, and increased portal pressure from chronic liver disease with portal vein thrombosis were suspected as possible causes of chylothorax in this patient. Further antegrade direct intranodal fluoroscopic lymphangiography was performed by an interventional radiologist, which revealed a leak at the T4-T5 vertebral level. Thoracic duct embolisation using 50% glue was carried out successfully (Figure 4).

For the multi-thrombotic state, tests for antinuclear antibody (ANA), anti-neutrophil cytoplasmic antibody (ANCA), antiphospholipid antibody (APLA), homocysteine, and thrombophilia panel were sent and came back negative.

Outcome

Patient was discharged on diuretics and oral anticoagulants. The serial follow-up chest X-rays showed minimal right pleural effusion with resolution of symptoms; however, the patient presented with chylous ascites on follow-up.

Final diagnosis in this case

Chylothorax resulting from multiple etiologies, which include thoracic duct leak, portal hypertension from chronic liver disease with portal vein thrombosis leading to chylous ascites tracking into the thoracic cavity, and thoracic duct obstruction at the veno-lymphatic junction due to subclavian vein thrombosis.

## Discussion

Chylothorax is a rare, potentially life-threatening condition. It is diagnostically challenging and requires careful distinction from other pleural effusions. Diagnostic criteria include milky pleural fluid with TGL levels >110 mg/dL, cholesterol <200 mg/dL, lymphocyte predominance, and chylomicron detection by lipid electrophoresis (if TGL levels are inconclusive) [4]. While typically exudative, transudative chylothorax is rare and is usually associated with cirrhosis, nephrotic syndrome, amyloidosis, and SVC obstruction [5].

Differential diagnoses are pseudochylothorax and empyema. Pseudochylothorax/cholesterol effusion is a condition where cholesterol ≥200 mg/dL, TGLs <110 mg/dL, cholesterol/TGL ratio >1, and no chylomicrons [6]. Empyema is a neutrophilic effusion, with positive culture, and clearing after centrifugation (unlike chyle, which remains milky).

Imaging

CT scan of the chest identifies associated masses or lymphadenopathy. MR lymphangiography can detect leaks from lymphatic disorders and malignancies. Conventional lymphangiography remains the gold standard for diagnosis and for enabling interventional procedures such as embolisation.

Treatment

Management mainly depends on the cause and rate of chylothorax collection. Usually, conservative approaches are used for low-output chylothorax (<1 L/day). A high-protein, low-fat diet with medium-chain TGLs is recommended. Medium-chain TGLs bypass the lymphatics, as they are directly absorbed by the intestine and transported to the liver through the portal vein. Supplementation with fat-soluble vitamins in low-output chylothorax is required. Medications like octreotide, somatostatin, and midodrine can also be used, as they reduce lymphatic flow by decreasing gastric, biliary, and pancreatic secretions and inhibit chyle absorption in the intestine [7,8]. In certain situations, total parenteral nutrition is used to reduce chyle production and support nutrition. In cases of high-output chylothorax (>1 L/day) that do not respond to conservative treatment, thoracocentesis, ICD, or indwelling pleural catheters offer symptomatic relief. Pleurodesis is indicated in refractory cases, but it is not appropriate for chylous ascites. Thoracic duct embolisation or ligation is necessary for anatomical causes resulting in persistent or high-output leaks.

Prognosis

Chylothorax carries a high risk of morbidity and mortality due to severe nutritional losses, immune compromise from lymphocyte depletion, and hemodynamic instability. In cirrhotic patients with chylous ascites, mortality may reach 40% [9].

## Conclusions

Chylothorax and chylous ascites are rare but clinically significant conditions with varied etiologies. While trauma is the most frequent cause, non-traumatic factors, such as malignancy, cirrhosis, and venous obstruction, should be carefully considered. Diagnosis is confirmed by pleural fluid and ascitic fluid analysis, and supported by imaging to localise the site of leakage. Management must be individualised. Low-output leaks may respond to dietary and pharmacological measures, whereas refractory or high-output cases require interventional or surgical procedures, such as thoracic duct embolisation. Multidisciplinary management and early recognition are essential to improve clinical outcomes.
